# The Role of Soluble CD163 (sCD163) in Human Physiology and Pathophysiology

**DOI:** 10.3390/cells13201679

**Published:** 2024-10-11

**Authors:** Andriana Plevriti, Margarita Lamprou, Eleni Mourkogianni, Nikolaos Skoulas, Maria Giannakopoulou, Md Sanaullah Sajib, Zhiyong Wang, George Mattheolabakis, Antonios Chatzigeorgiou, Antonia Marazioti, Constantinos M. Mikelis

**Affiliations:** 1 Laboratory of Molecular Pharmacology, Department of Pharmacy, University of Patras, 26504 Patras, Greece; andrianaandriana775@gmail.com (A.P.); margaritalamprou@upatras.gr (M.L.); elenimourk@upatras.gr (E.M.); up1068132@ac.upatras.gr (N.S.); marakig2001@gmail.com (M.G.); 2 Department of Pharmaceutical Sciences, School of Pharmacy, Texas Tech University Health Sciences Center, Amarillo, TX 79106, USA; sajib1989@gmail.com; 3 Stomatology Hospital, School of Stomatology, Zhejiang University School of Medicine, Clinical Research Center for Oral Diseases of Zhejiang Province, Key Laboratory of Oral Biomedical Research of Zhejiang Province, Cancer Center of Zhejiang University, Hangzhou 310006, China; wzy0809@zju.edu.cn; 4 School of Basic Pharmaceutical and Toxicological Sciences, College of Pharmacy, University of Louisiana Monroe, Monroe, LA 71201, USA; 5 Department of Physiology, Medical School, National and Kapodistrian University of Athens, 75 Mikras Asias Str., 11527 Athens, Greece; achatzig@med.uoa.gr; 6 Basic Sciences Laboratory, Department of Physiotherapy, School of Health Sciences, University of Peloponnese, 23100 Sparta, Greece; a.marazioti@uop.gr

**Keywords:** CD163, soluble CD163, sCD163, endothelial cells, physiology, cancer

## Abstract

Soluble CD163 (sCD163) is a circulating inflammatory mediator, indicative of acute and chronic, systemic and non-systemic inflammatory conditions. It is the cleavage outcome, consisting of almost the entire extracellular domain, of the CD163, a receptor expressed in monocytic lineages. Its expression is proportional to the abundance of CD163^+^ macrophages. Various mechanisms trigger the shedding of the CD163 receptor or the accumulation of CD163-expressing macrophages, inducing the sCD163 concentration in the circulation and bodily fluids. The activities of sCD163 range from hemoglobin (Hb) scavenging, macrophage marker, decoy receptor for cytokines, participation in immune defense mechanisms, and paracrine effects in various tissues, including the endothelium. It is an established marker of macrophage activation and thus participates in many diseases, including chronic inflammatory conditions, such as atherosclerosis, asthma, and rheumatoid arthritis; acute inflammatory conditions, such as sepsis, hepatitis, and malaria; insulin resistance; diabetes; and tumors. The sCD163 levels have been correlated with the severity, stage of the disease, and clinical outcome for many of these conditions. This review article summarizes the expression and role of sCD163 and its precursor protein, CD163, outlines the sCD163 generation mechanisms, the biological activities, and the known underlying molecular mechanisms, with an emphasis on its impact on the endothelium and its contribution in the pathophysiology of human diseases.

## 1. Introduction

**Figure 1:**
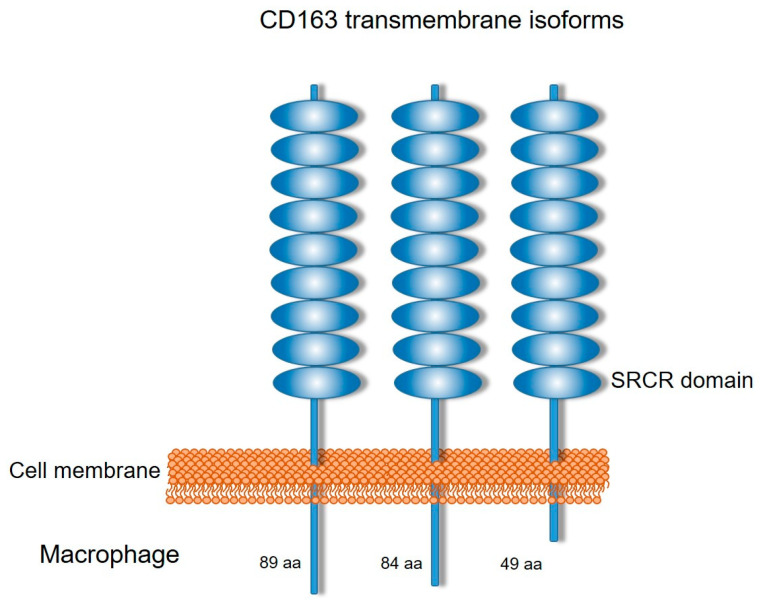
Schematic representation of the three CD163 transmembrane isoforms. They differ in the length of the intracellular domains, with the 49-amino-acid isoform having dominant expression.

Macrophages are important regulators of homeostasis, in physiological and pathological conditions. They contribute to tissue morphogenesis and repair, regulating inflammatory processes, and can be found either in the bloodstream or tissues, contributing to host defense and inflammation evolution. This is due to their ability to phagocytose, degrade self and foreign materials, and produce and respond to inflammatory mediators. Macrophages in the blood stream originate from myeloid progenitor cells in the bone marrow, differentiated into monocytes, while the tissue-resident ones are derived from yolk sac or fetal liver precursors [1,2]. Macrophages recognize a variety of ligands and eliminate them via pattern recognition, a process mediated by Pattern Recognition Receptors (PRRs). Scavenger receptors (SRs) are a subgroup of membrane-associated PRRs. SRs are cell-surface phagocytic receptors that bind to either damage-associated molecular patterns (DAMPs), such as modified low-density lipoprotein (LDL), or pathogen-associated molecular patterns (PAMPs). SRs have diverse structural traits and biological function and are divided into different classes, depending on common ligands and structural determinants [3,4]. CD163 is a membrane receptor for haptoglobin–hemoglobin (Hp–Hb) complexes, expressed in monocytes and macrophages [5]. It was initially considered a member of the B group of the scavenger receptor cysteine-rich (SRCR) family [6], but now it belongs to Class I1 of SRs (SR-I1), which corresponds to scavenger receptors that are type I transmembrane receptors characterized by multiple ancient extracellular SRCR domains [3,4,7]. It also actively participates in host defense mechanisms due to its ability to recognize pathogens, as it has been shown to bind bacteria and viruses [8,9,10,11,12]. CD163 is a 130 kDa glycoprotein that contains a single transmembrane domain, a short cytoplasmic tail, and a large extracellular region with nine SRCR domains [13]. Three splice variants have been reported, with different cytoplasmic tail lengths, two long and one shorter tail form [6,13,14] (Figure 1). The short-tailed isoform presents the most pronounced surface expression, with the highest capacity for ligand endocytosis [15]. Soluble CD163 (sCD163) is a soluble inflammatory mediator that is derived from enzymatic cleavage of CD163 [5]. Due to the macrophage-specific expression of the precursor protein, CD163, the expression and upregulation of sCD163 are directly related to CD163 expression and activation levels of CD163-expressing macrophages. Given the consideration of sCD163 as a marker for several conditions, the aim of the present article was to summarize what is known about the involvement of sCD163 in these conditions and to identify the missing pieces in the puzzle of biological activity and therapeutic potential. Thus, the criteria for the selection of literature included the mechanisms of sCD163 generation and level regulation, the biological activity and the molecular mechanisms at steady state, and pathological conditions where the sCD163 are upregulated.

## 2. CD163 Expression

CD163 was initially identified as the antigen of four macrophage/monocyte-targeting monoclonal antibodies: Ki-M8, Ber-MAC3, GHI/61, and SM4 [13], revealing its immune cell-specific expression pattern. CD163 mRNA expression has been detected by Northern blot analysis in the spleen, lymph nodes, thymus, and fetal liver. Low levels of CD163 mRNA were identified in bone marrow, and no expression was detected in peripheral blood leukocytes [6]. CD163 is highly expressed in most tissue-specific macrophages and is present with lower expression in monocytes [13,16,17]. In fact, CD163 expression in monocytes is limited (5–30%), while it is more prominent in subpopulations of mature tissue macrophages [6,18], thus is now considered a differentiation marker. CD163 is detected in macrophages located in various organs, such as Kupffer cells in the liver, red pulp macrophages in the spleen or alveolar macrophages, thymus, tonsils, perivascular and meningeal macrophages, dermal macrophages (skin), and lymph nodes [18].

Macrophages are activated via stimuli-dependent processes, and the broad classification of macrophages upon activation is pro-inflammatory (M1) and anti-inflammatory (M2) macrophages. However, this categorization is incapable of describing intermediate phenotypes with mixed characteristics or the phenotypic adaptation as a result of microenvironmental stimuli [1]. Although CD163 has been considered a classic M2 macrophage marker [19], there are conflicting reports on whether CD163 expression per se determines such a polarization. While CD163 and CD206 are considered M2 macrophage markers [20], it has been reported that the CD163 expression alone cannot determine macrophage polarization; instead, the combination of other markers, such as pSTAT1 and RBP-J or CMAF, should be used to determine M1 or M2 polarization, respectively [21].

Aligned with its role on the polarization of macrophages, CD163 is mostly considered an M2 macrophage marker [19] and is targeted via diverse mechanisms, including microRNAs. MicroRNAs (miRNAs) are small fragments of non-coding RNA that regulate gene expression and protein synthesis. They control cellular functions such as differentiation, development, and apoptosis [22]. miR-106b-5p secretion from glioma cells promotes M2 polarization and CD163 upregulation via inhibition of the IRF1/IFN-β pathway [23]. Two different miRNAs, miR-181c and miR-125a-5p, target CD163 expression via independent mechanisms: miR-181c targeted CD163 mRNA for degradation, while miR-125a-5p inhibited CD163 expression inducers, such as IL-10 signaling, eliciting a pro-inflammatory response [24]. Sulfatase 2 promoted bladder cancer development by promoting M2 macrophage polarization via CD163 upregulation downstream of the JAK2/STAT3 pathway [25]. Thus, targeting tumor-associated macrophage (TAM) polarization through CD163 expression has been suggested as a therapeutic approach for macrophage-dependent diseases, such as infections and malignancies [25,26].

**Figure 2:**
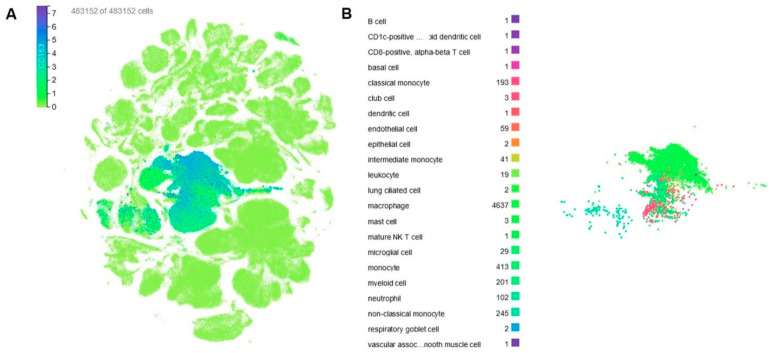
(**A**) Uniform manifold approximation and projection (UMAP) representation of all human cell types based on CD163 expression (data from 483,152 cells from the CZ database). (**B**) Clustering and analysis of the cell types with the highest CD163 expression.

The accumulation of single cell sequencing databases allows the display of the cell-specific expression of the target gene [27]. A detailed search for CD163 cell-specific expression using the CZ CELLXGENE database [28,29,30] confirmed the CD163 expression by macrophages and monocytes (Figure 2A). Selection of the CD163-expressing cells with the highest expression (using a threshold of 4) revealed some expression by myeloid cells and neutrophils (Figure 2B), while in terms of organs at steady state, the vasculature, lung, adipose tissue, bladder, and spleen present higher expression, which should correlate with macrophage abundance in each organ.

## 3. Role of CD163

**Figure 3:**
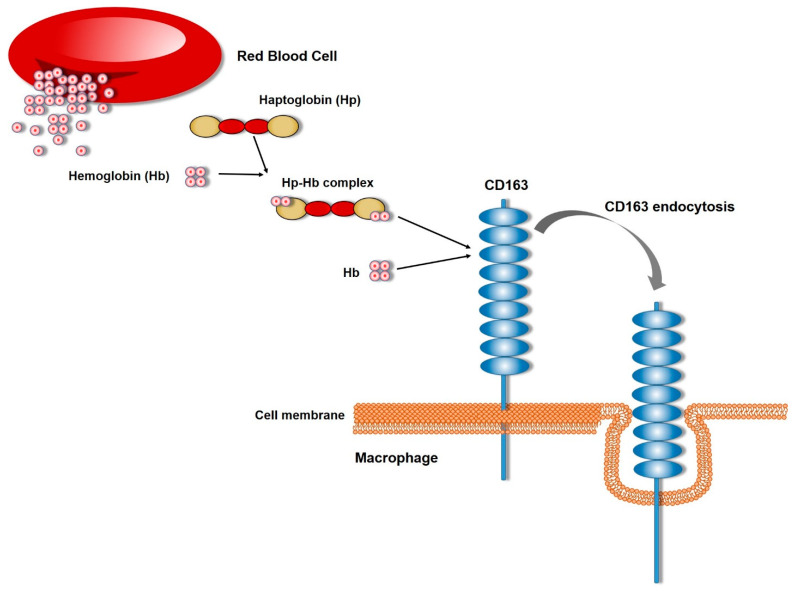
Schematic representation of the hemoglobin clearance mechanism by CD163 in inflammatory conditions. The Hb released by the ruptured red cells is bound to the 3rd SRCR domain of the CD163 extracellular domain, either by itself or as a Hp–Hb complex, which eventually leads to CD163 internalization.

One of the first functions attributed to CD163 was hemoglobin scavenging via the endocytosis of the Hp–Hb complexes. Macrophages protect the tissues from oxidative damage caused by iron-containing heme in cases of either extravascular or intravascular hemolysis, especially in cases of infection and autoimmune disorders. They have the ability to engulf senescent erythrocytes or take up released hemoglobin (Hb) from ruptured erythrocytes and immature erythrocytes in the bone marrow and metabolize heme to bilirubin, iron, and carbon monoxide (CO) by heme oxygenase (HO) enzymes. Haptoglobin (Hp) is an abundant plasma protein that binds to Hb, promoting its clearance [31] (Figure 3). Hp is depleted during elevated hemolysis [18], thus it was hypothesized that Hp mediated the mechanism of Hb clearance. CD163 was identified as the responsible receptor for Hb scavenging by macrophages when it was isolated from a Hp–Hb affinity matrix with affinity chromatography of solubilized membranes from three macrophage-containing human tissues [32]. The interaction between CD163 and Hp–Hb complex was of high affinity and was regulated by the type of Hp–Hb complex [32] and the Ca^2+^ levels [32,33], while it was shown that the SRCR domain 3 was the one responsible for this function [33]. Not long later, it was identified that CD163 can interact and scavenge Hb in the absence of Hp. In fact, Hb can bind to CD163, leading to receptor-dependent endocytosis, which also inhibits Hp–Hb endocytosis, suggesting a common binding site [34] (Figure 3). CD163 expression in different organs has been correlated with this primary function, such as its expression in the liver, in Kupffer cells, the resident macrophages of the liver, where it plays a role in hemoglobin clearance and immune regulation [35].

The scavenging effect of CD163 on “free” Hb is known to play a vital role in hematoma absorption in patients with intracerebral hemorrhage (ICH), where spontaneous, non-traumatic bleeding leads to hemoglobin release with damaging potential to the surrounding tissues. CD163 protects against this damage by binding to free hemoglobin and facilitating its removal. It was shown that patients with higher concentrations of soluble CD163 presented increased hematoma absorption and improved neurological deficits. Thus, CD163 was proposed as a target for treatment of intracerebral hemorrhage [36]. It was further identified that CD163 was responsible for the improved hematoma resolution effect by CCL17, which included activation of the C-C chemokine receptor 4 (CCR4) with downstream ERK and Nrf2 activation, improving the outcome of intracerebral hemorrhage in a relevant animal model [37]. The role of CD163 (including sCD163) in hemoglobin clearance in hemorrhage has been extended in other tissues, such as in hepatic hemorrhage [38].

CD163 has an established role in host defense mechanisms. Experimental data support its function as a PRR since Chinese Hamster Ovary (CHO) cells expressing CD163 had the ability to bind Gram-negative and Gram-positive bacteria via a motif localized on the SRCR domain. Moreover, bacterial recognition by CD163 was found to potently enhance inflammatory cytokine production in monocytic THP-1 cells [12]. CD163 acts as a receptor for Porcine Reproductive and Respiratory Syndrome Viruses (PRRSVs) and is responsible for susceptibility to PRRSV into non-permissive cells, regulating viral entry, while CD163-deficient animals are resistant to PRRSV infection [8,9,10,11]. Moreover, Siglec1 and CD163 have been reported to act synergistically as receptors for African swine fever virus invasion of host cells, although the mechanism has not yet been delineated [39].

The expression of CD163 by macrophages has been associated with inflammation regulation since its expression is induced by anti-inflammatory cytokines and its functionality in terms of Hb scavenging results in the production of the anti-inflammatory heme metabolites [1]. Proinflammatory cytokines, such as IFN-γ or TNF-α, decrease CD163 expression. The same effect was observed after treatment with lipopolysaccharide (LPS), which depends on the examined dose. On the other hand, anti-inflammatory cytokines IL-10 and IL-6 induce CD163 expression [40]. Strong induction of CD163 mRNA and protein expression has been described in vitro and in vivo by glucocorticoids [40,41,42]; as an example, treatment of monocytes with dexamethasone, apart from triggering CD163 expression, also increased adhesion to unstimulated and stimulated human umbilical vein endothelial cells (HUVECs) [41]. The mechanism of IL-10-driven increased expression of CD163 is independent from the glucocorticoid-driven induction [43,44]. At the same time, the CD163 anti-inflammatory effect is reported to be mediated, at least partly, by IL-10 secretion, which was triggered from the binding of Hp–Hb complexes and the subsequent heme oxygenase-1 stress protein synthesis. The phenomenon was abolished upon treatment with antibodies against CD163 or IL-10 [45]. Contrary to the aforementioned cytokines and agents that induce CD163 expression, several anti-inflammatory cytokines, such as IL-4 and IL-13 [43,44], immunosuppressants cortisol and cyclosporine A [40], or indomethacin [42], do not induce CD163 expression.

A noteworthy interaction of CD163 is with tumor necrosis factor-like weak inducer of apoptosis (TWEAK). TWEAK is a member of the TNF superfamily that regulates a wide range of cellular processes, such as proliferation, migration, differentiation, apoptosis, angiogenesis, and inflammation [46]. It exerts its effects via various intracellular signaling pathways, including NF-kB, ERK/MAPK, Notch, EGFR, and AP-1. In healthy tissues, TWEAK signaling has protective effects, but in chronic inflammatory conditions it can become harmful. Two receptors have been characterized for TWEAK: Fn14, which binds to the membrane-bound form of TWEAK, and CD163, which clears the soluble form of TWEAK (sTWEAK) from circulation. CD163 binds and internalizes sTWEAK, regulating NF-kB and Notch activation. sTWEAK has a beneficial role in ischemia, as it stimulates myogenic progenitor cell proliferation, promoting muscle repair, while low levels of circulating sTWEAK have been associated with worse cardiovascular outcomes in both diabetic and non-diabetic individuals. TWEAK seems to trigger distinct cellular events, depending on the receptor it binds, the tissue it is expressed in, external conditions, and the presence of other cytokines. Strong evidence supports the involvement of the TWEAK/Fn14/CD163 axis in metabolic disorders, chronic autoimmune diseases, and acute ischemic stroke [47].

CD163 binding by a specific monoclonal antibody increased the secretion of IL-1b, IL-6, and GM-CSF cytokines. The molecular cues regulating the secretion of each cytokine are not identical, as the protein tyrosine kinase (PTK) inhibitor genistein blocked IL-6 and GM-CSF production but not of IL-1b [18,48].

In terms of downstream signaling targets of CD163, casein kinase II (CKII) and PKC have been highlighted. The regulatory β-subunit of CKII interacts with the cytoplasmic domain of all three CD163 isoforms, while both PKC-a and CKII phosphorylate CD163 in vitro. The inhibition of either CKII or PKC reduced the IL-6 and IL-1β secretion [48]. The CD163 phosphorylation is not implicated in Hb endocytosis or the subsequent HO-1 induction, since a mutated form of the CD163 receptor, in which all phosphorylation sites were removed, was able to internalize the Hp–Hb complex [49].

## 4. Generation of sCD163

**Figure 4:**
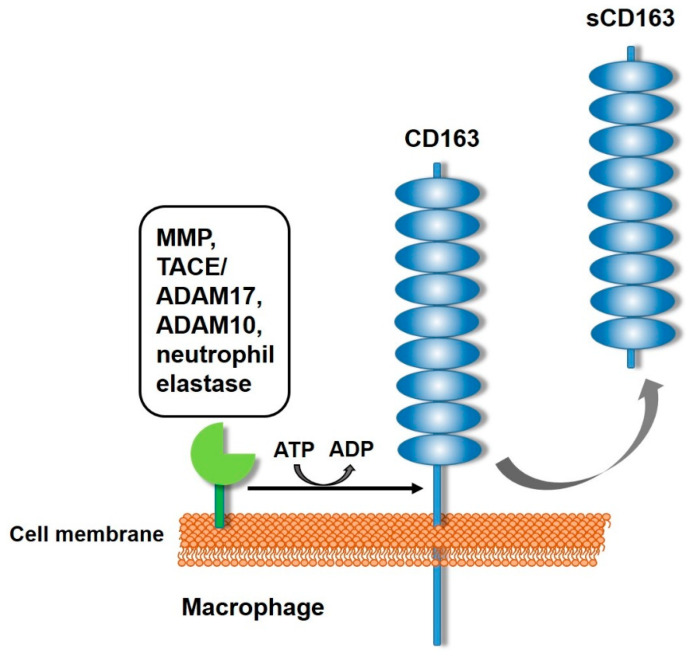
Schematic representation of sCD163 generation from the proteolytic cleavage of CD163. The list of the proteases known to cleave CD163 is shown on the left and the cleavage requires ATP consumption (black arrow).

As initially mentioned, sCD163 consists of the extracellular region of the CD163, derived upon proteolytic cleavage. The proteolytic cleavage is enhanced by inflammatory responses or oxidative stress, resulting in the generation of sCD163 and, therefore, its release in the plasma and other tissue fluids [20]. The proteolytic cleavage takes place near the cell membrane, thus sCD163 maintains almost the entire extracellular part of CD163, corresponding to 945 amino acids (94% of the extracellular part) and including all nine extracellular SRCR domains, as verified by mass spectrometry of human serum-derived sCD163 [5,50] (Figure 4).

The CD163 ectodomain is being shed from the cell surface of macrophages by proteolytic cleavage upon matrix metalloproteinase (MMP) activation, specifically tumor necrosis factor α-converting enzyme/A disintegrin and metalloprotease domain 17 (TACE/ADAM17) [51,52]. More than 40 membrane-bound protein substrates have been characterized as ADAM17 shedding targets, including TNF-α, which can explain the simultaneous shedding of both mediators [52,53]. LPS can activate ADAM17, an inflammation-responsive protease that cleaves extracellular domains of transmembrane CD163. The shedding mechanism is ATP-mediated, and similar results have been reported with PMA stimulation (phorbol 12-myristate 13-acetate), which activates protein kinase C and the production of a variety of cytokines [51,54,55]. Consequently, ligands of Toll-like receptors (TLRs) 2, 4, 5, and immunologic stimulants can be the aftermath of increased levels of sCD163 in the plasma. An alternative route could be crosslinking of the Fcγ receptor in monocytes, as this receptor plays a crucial role in the endocytosis of hemoglobin and, accordingly, in the homeostasis of CD163 [5]. ADAM 10, along with ADAM17, has been reported to induce shedding of CD163 upon *Staphylococcus aureus* and *Staphylococcus pyogenes* infection [56]. Lastly, neutrophil elastase, a serine proteinase that targets bacteria during inflammation, has also been reported to shed CD163 on monocytes [57].

## 5. sCD163 Activities

The primary function attributed to sCD163, similar to CD163, is the absorption and elimination of unbound hemoglobin, thereby reducing oxidative damage caused by heme radicals, since free hemoglobin in the bloodstream can contribute to oxidative stress and tissue damage. The scavenging function of sCD163 helps mitigate these effects. It has been demonstrated that sCD163 and immunoglobulin G interact with the free Hb in plasma, leading to monocytic endocytosis of the sCD163-Hb-IgG complex via the Fcγ receptor (FcγR) [58]. Hence, the balance between CD163 expression and circulating sCD163 is restored on the monocytes, with the internalized Hb being catabolized as well [58]. However, it has been demonstrated that Hp–Hb “prefers” binding to the transmembrane form of CD163 rather than its soluble form, where it needs a large excess of Hp–Hb complex to bind. The preference for the membrane-bound form of CD163 can be explained based on the structure of the Hp–Hb complex, which theoretically enables cross-linking of two or more receptors via Hp. Hp has two Hb-binding sites in the Hp 1-1 phenotype and multiple binding sites in the Hp 2-1 and 2-2 phenotypes, despite the optimal Hb binding on the Hp 1-1 phenotype. This cross-linking likely stabilizes the binding, making it far less prone to dissociate compared to the binding of Hp–Hb to soluble CD163 [59,60].

sCD163 is a marker of activated macrophages, and its role is to modulate inflammatory reactions. Ordinarily, it shields monocytes from hyperactivation during infectious and inflammatory diseases by reducing the excretion of proinflammatory cytokines, such as TNF-a, IL-1β, IL-6, and IL-8 [61]. It can also suppress the production of pro-inflammatory chemokines, such as monocyte chemoattractant protein-1 (MCP-1), while promoting the secretion of anti-inflammatory mediators, such as interleukin-10 (IL-10) [62].

The activities of sCD163 are largely regulated by its interactions with protein targets, and sCD163 can interact with other receptors and molecules, influencing various cellular processes. A characteristic example is the interaction with TWEAK. As mentioned above, TWEAK is a cytokine that regulates inflammation, cell growth, angiogenesis, and also apoptosis under certain conditions [46]. CD163 is one TWEAK receptor, mediating its downstream functions. CD163 scavenges the soluble form of TWEAK, and receptor Fn14 targets its membrane form. sCD163 also interacts with TWEAK and serves as a decoy receptor, controlling TWEAK-induced Notch activation and muscle regeneration. Mice lacking CD163 expression transiently expressed higher TWEAK levels and enhanced muscle regeneration [63]. On the contrary, increased sCD163 levels lead to decreased TWEAK levels, while CD163 deficiency triggers plaque formation in atherosclerotic mice, suggesting the sCD163/TWEAK plasma ratio as an atherosclerosis marker [20,64]. In a similar notion, CD163-TWEAK interaction seems to play a role in Systemic Sclerosis pathogenesis, and high sCD163 levels were shown to have a protective role against ulcer development in Systemic Sclerosis patients [65]. sCD163 interacts with extracellular matrix (ECM) proteins, such as fibronectin (FN), used by pathogens to adhere to the target cells. Thus, as part of the immune defense mechanism, sCD163 specifically binds to *S. aureus* and *S. pyogenes* via the Fibronectin binding protein (FnBP) adhesin, acting as an opsonization factor [56].

Apart from the aforementioned TWEAK-based activities, there is further evidence highlighting the relationship between sCD163 and endothelial functions. CD163-deficient mice presented reduced angiogenesis, associated with decreased bone volume in a bone fracture repair model [66]. The endothelial progenitor cells (EPCs) represent a subset of peripheral blood mononuclear cells (PBMNCs) that can differentiate in vivo into endothelial cells, thus consisting of a promising cell type for regenerative therapeutic approaches. EPCs have a higher potency to promote neovascularization after ischemia than mature endothelial cells [67,68]. CD163 was included in the macrophage marker group expressed in EPCs and sCD163 was identified in their conditioned medium [68], suggesting that CD163 may be associated with endothelial differentiation and function. The association between sCD163 and endothelial functions is highly dependent on the angiogenic potential of CD163-expressing macrophages. High CD163 expression has been associated with poor prognosis in classical Hodgkin’s Lymphoma, since its expression was analogous to vascular endothelial growth factor (VEGF) expression and tumor microvascular density, while high CD163 expression was associated with decreased event-free survival and overall survival of Hodgkin’s Lymphoma patients [69]. As a TAM marker, CD163 was positively correlated with microvascular density, lymph node metastasis, and lymphovascular invasion in gastric cancer. Mechanistically, this was associated with CXCL12 abundance due to its involvement in TAM distribution in this tumor [70]. Elimination of CD163-expressing TAMs triggered T cell infiltration and tumor regression, implying the role of paracrine TAM-derived tumor-promoting mechanisms [71]. However, the topic requires further assessment, with a focus on the role of sCD163 in the tumor microenvironment. Macrophage-driven angiogenesis is not restricted to tumors; the abundance of the CD163^+^ macrophages was shown to promote angiogenesis and vascular permeability in other inflammatory conditions, such as atherosclerosis via HIF1α activation and VEGF expression [72].

## 6. sCD163 as a Biomarker and Role in Pathological Conditions

sCD163 stands out as a valuable biomarker, commonly utilized alongside others in various studied cases, and numerous reports have correlated the progression or the severity of several pathological conditions with its levels in tissue fluids, serum, or plasma. sCD163 is recognized as a biomarker of macrophage activation and systemic inflammation [5]. Due to the macrophage participation in the pathophysiology of systemic and non-systemic diseases, it is logical that sCD163 levels in plasma or biological fluids have been investigated for diagnostic and prognostic purposes [73,74,75]. sCD163 levels have been linked with a number of chronic inflammatory conditions, such as atherosclerosis, asthma, and rheumatoid arthritis [1]. Atherosclerosis is a common inflammatory disease, where sCD163 levels and the sCD163/TWEAK plasma ratio are proposed as atherosclerotic markers [64,76,77]. As in the case of atherosclerosis, also in tissue ischemia, sCD163 functions as a decoy receptor for TWEAK, regulating its ability to activate the Notch signaling pathway and leading to myogenic progenitor cell proliferation [63]. In asthma, sputum, and serum, sCD163 levels were inversely correlated with lung function [78], while in rheumatoid arthritis, sCD163 levels in serum and synovial fluid were elevated and considered a reliable marker of macrophage activation [79]. Tumors are considered chronic inflammatory conditions, and sCD163 levels have been evaluated for prediction and diagnostic purposes in several tumor types [80,81]. More information is included in the tumor-specific section.

Higher sCD163 serum levels have also been identified in acute inflammatory conditions, such as sepsis [82], hepatitis [83] and malaria [84]. Sepsis is a disease characterized by generalized inflammation. In sepsis, the serum sCD163 levels provide diagnostic value and correlate with the severity of the condition. The value of sCD163 was higher than of other potential markers tested, such as procalcitonin and C reactive protein [82]. Macrophage-derived mediators seem to be involved in hepatitis, with serum sCD163 levels demonstrating a positive correlation with the disease progression and survival [83], while sCD163 levels in malaria were increased, suggesting inflammatory dysregulation [84]. Higher sCD163 serum levels have been identified in other inflammatory conditions, such as hemophagocytosis [85]. For more information on this topic, the reader is referred to an excellent review article [86].

Exogenous ligands of CD163 are bacteria [12] and viruses [87], which activate the macrophages during bacterial and viral infections, respectively. Increased sCD163 levels, as a macrophage activation marker, have been reported for human immunodeficiency virus (HIV), dengue, influenza, hepatitis B and C, Epstein–Barr viruses, and measles, to name a few, the clinical value of which has been reported elsewhere [88]. In HIV, higher levels of sCD163 are associated with viral activity, inflammation, and a greater mortality risk, particularly among those on antiretroviral therapy (ART). In Dengue, elevated sCD163 levels indicated more severe cases, including its possible role in hemophagocytic syndrome. In COVID-19, this biomarker is related to more severe outcomes, such as respiratory distress syndrome (ARDS), and in influenza, it has been linked to complications like encephalopathy [88]. COVID-19 patients presented higher sCD163 serum levels, with a significant difference for the intensive care unit (ICU)-admitted patients [75]. Anakinra treatment decreased sCD163 levels, but this change did not determine the clinical outcome [89]. sCD163 also tracks liver inflammation and fibrosis in hepatitis B and C infections, as well as renal failure in Hantaan virus infection. Moreover, elevated sCD163 levels are observed in Epstein–Barr and measles virus infections, indicating its broader relevance in tracking viral disease severity [88]. However, despite the use of sCD163 levels as a macrophage activation marker, its specific function in these diseases is not known. As a conclusion, sCD163 seems to be a promising marker for assessment of disease progression and outcomes in viral infections, though further validation is required to enhance its clinical use.

sCD163 levels, as a means for macrophage activation, have been associated with insulin resistance and the development of type 2 diabetes [90,91,92], obesity [73], lipid metabolism, and type 1 diabetes [93]. For liver diseases, sCD163 has been investigated as a potential biomarker for both acute and chronic liver diseases, with sCD163 abundance correlating with disease severity [74,94]. sCD163 levels are associated with morphological features of non-alcoholic fatty liver disease (NAFLD), non-alcoholic steatohepatitis (NASH), and liver fibrosis [95]. On the other hand, prolonged physical activity also leads to increased sCD163 levels, which are attributed to the increased presence of CD163^+^ macrophages upon exercise-induced pro-inflammatory effects. This shows that certain amounts of sCD163 are important for a healthy balance and could be a pathway through which exercise can exert its beneficial effects [96]. Below, we have compiled a table (Table 1), summarizing all known pathological cases where the levels of sCD163 are impacted.

## 7. sCD163 in Tumors

The role of sCD163 in tumors has been extensively explored the last decade, partly due to the interest in macrophages as a target for immunotherapy. sCD163 seems to be elevated in patients with inflammatory diseases as well as with several tumor types. Its presence has been widely reported in different types of tumors, and, commonly, higher levels are associated with worse clinical outcomes [1].

In tumors, TAMs, among other immunosuppressive cells, such as myeloid-derived suppressor cells, regulatory T cells, and tumor-associated neutrophils, promote tumor growth by producing chemokines and angiogenic factors. Thus, as a general notion, the presence of TAMs indicates a poor prognosis. TAMs support tumor initiation, tumor progression, and metastasis by promoting biological processes such as angiogenesis, immunosuppression, and proliferation of tumor cells, and their contribution to the tumor microenvironment for prognostic purposes has been established [1,101]. The anti-inflammatory M2 macrophages drive immunosuppression and favor tumor growth via the production of factors such as VEGF, IL-6, IL-10, CXCL5, CXCL10, and MMPs. CD163 expression by monocytes and macrophages indicates reduced inflammation, along with high CD163 levels in TAMs, contributing to elevated sCD163 levels [102]. It is noteworthy that in the serum of tumor patients, sCD163 levels are elevated, thus measuring its levels in the peripheral blood could be an indicator of the total-body M2 macrophage load. Moreover, there is an increasing number of studies showing that elevated levels of sCD163 in the serum are linked to poor prognosis in multiple types of tumors [81]. Therefore, CD163^+^ TAMs have been proposed as tumor biomarkers, while their potential as targets for anti-cancer immunotherapeutic approaches is high and worth exploring. Since sCD163 levels go hand in hand with CD163 expression, we highlight below tumor types where such a role for CD163 or sCD163 has been explored or considered.

Breast cancer: Being the most frequent malignant tumor in women, the role of TAMs in breast cancer has been explored, and they are associated with a poor prognosis. CD163 has dethroned the earlier considered pan-macrophage marker CD68 as a better predictor for poor survival of breast cancer patients. In a comparative study, the CD163^+^ TAM density was better associated with worse clinical outcomes than that of the CD68^+^ TAMs [103]. Interestingly, the higher CD163^+^ TAM density in the tumor stroma compared to the tumor area was associated with a decreased survival rate, showing a strong tumorigenic function of this subset of TAMs [104].

Head and neck cancer: Head and neck squamous cell carcinomas (HNSCC) are considered immunogenic; thus, the tumor microenvironment demonstrates a regulatory role, with TAMs being a major component. As an M2 macrophage marker, CD163^+^ TAMs have been correlated with worse survival, and CD163 has been proposed as a prognostic biomarker. Like in the case of breast cancer, the abundance of CD163^+^ TAMs was more predictive for overall and progression-free survival than of the CD68^+^ TAMs [105]. Overall, a lower number of CD163^+^ TAMs in tumor patients correlated with a higher survival rate [105,106]. The selective CD163 expression on monocytic lineages (monocytes, macrophages, and dendritic cells) would provide a reliable prognostic marker and therapeutic target for HNSCC [105,106].

Pancreatic cancer: In pancreatic ductal adenocarcinoma (PDAC), high CD163 expression, as a marker for M2 macrophages, was inversely correlated with overall survival [107]. A recent study evaluated the diagnostic and prognostic potential of sCD163 in PDAC patients. sCD163 levels of the PDAC patients were significantly elevated than these of the healthy subjects, and sCD163 in combination with CA19-9, a pancreatic cancer marker, offered higher predictive potential than sCD163 alone [108]. Although further studies are required to verify the prognostic and diagnostic value of plasma sCD163 in pancreatic tumors, based on the so far reports, the potential is high.

Sarcoma: Soft tissue sarcoma (STS) remains a therapeutic challenge, as it does not follow the improvement of the overall survival that takes place in other tumors with advanced therapeutic approaches. The current treatment remains adjuvant chemotherapy, but the responders remain a small proportion, plus a stratification method to select these patients is missing. Moreover, there is a limited response to checkpoint inhibitors due to the non-immunogenic nature of these tumors. A recent study showed elevated levels of sCD163 in serum from patients with STS, and it was correlated with increased disease recurrence and poor overall survival [109].

Lung cancer: Contrary to sarcomas, lung cancers are considered immunogenic but characterized by high heterogeneity. Also in these tumors, CD163 expression was assessed as an M2 macrophage marker, and high M2 macrophage abundance was correlated with high metastatic incidence and poor survival. In non-small cell lung cancer, the abundance of CD163^+^ TAMs was associated with the tumor stage, invasive size and differentiation, proliferation rate, and the presence of lymph node metastases, with C-reactive protein (CRP) levels being used as a serum marker. However, the CD163^+^ TAM distribution is important for disease free survival and overall survival assessments, as the stromal and alveolar CD163^+^ TAMs were significantly associated with these parameters, but the islet ones were not [110,111]. Moreover, although CD163^+^ macrophages were identified in both malignant pleural effusion and bronchoalveolar lavage fluid (BALF) of lung cancer patients, only the levels of the malignant pleural effusion ones were significantly inversely correlated with progression-free survival, thus offering prognostic value [112,113]. In lung adenocarcinoma, high CD163 expression was correlated to decreased progression free and overall survival, while a similar trend took place for the squamous cell carcinoma cases [114]. In the same line, in breast cancer it has been reported that CD163 expression is not only confined to macrophages but can also be expressed by cancer cells [115].

Colorectal cancer: High CD163 expression at the tumor invasive front was correlated with high tumor grade, invasion, the presence of lymph node metastases, and low overall survival [110,116]. In a Swedish patient cohort, the abundance of CD163^+^ TAMs in biopsies was correlated with earlier recurrence and poor survival [117], with contradicting results in a Greek patient cohort, where lower tumor grade, fewer lymph node metastases, and improved survival were correlated with increased stromal infiltration of CD163^+^ TAMs [118].

Bladder cancer: Like in other cancer types, also in bladder cancer, CD163^+^ TAM infiltration was analogous to histologically advanced disease. It is worth noting that CD163 expression was not confined to macrophages but took place in malignant cells, which could be associated with epithelial-to-mesenchymal transition of the tumor cells and should contribute to the overall patient outcome [119]. The ectopic expression of CD163 on cancer cells due to cell fusion has been experimentally validated [119], has been reported in several cancer types [115,120,121] and seems like a common and valid mechanism that needs to be considered in the design of anti-cancer immunotherapeutic approaches.

Overall, CD163 in tumors has been widely used as a TAM marker, especially of the M2 orientation, and its high expression levels have been correlated with poor prognosis, metastasis, and overall survival in various cancers. The immunosuppressive role of the CD163^+^ macrophages in tumors highlights the anti-inflammatory role of the tumor microenvironment, which leads to tumor growth and poor clinical outcomes. The angiogenic impact of the CD163^+^ macrophages on the vasculature of the tumor microenvironment leads to increased oxygenation and nutrition of the tumoral area, contributing to the final clinical outcome. While the underlying mechanisms have started to be delineated [1], more work is required. The abundance of the CD163^+^ macrophages leads to the proportional elevation of the sCD163 levels in the serum of cancer patients [122]. However, whether sCD163 has a direct function in cancer development impacting the tumor microenvironment or its expression levels serve only as a prognostic marker needs to be elucidated.

## 8. Conclusions

Circulating sCD163 serves as a biomarker of systemic or non-systemic inflammatory conditions. The role of sCD163 in several diseases, including neoplasms, has been studied, and there are many reported suggestions of its potential as a biomarker for the presence and severity of diseases. Its presence highlights the expression of the precursor transmembrane protein CD163, which is expressed mostly in monocytes and macrophages. The CD163 cleavage and sCD163 production are triggered by known molecules, such as MMPs and ADAM17, with the strong possibility that more mechanisms will be identified in the future. There are pieces in the puzzle of the role of sCD163 missing, especially regarding its impact in the tumor microenvironment and in other tissues and organs, while ectopic expression of its precursor protein, CD163, from tumor cells needs to be taken into consideration. An important question that has started being delineated is the functional role of the soluble receptor. While it has been shown to not have sufficient antagonistic activity for Hb clearance due to the limited Hp–Hb binding efficiency, its role in other settings is not known, and our view leans toward antagonistic function at least in some settings. The continuous, intense scientific efforts are expected to reveal its functions along with the associated molecular mechanisms and provide an important diagnostic tool and target for therapeutic approaches.

## Figures and Tables

**Table 1:** Known pathological conditions where the levels of sCD163 have been reported to be elevated [1,88,97,98,99,100].

Pathological Conditions Where Elevated sCD163 Levels Have Been Reported	
Acute Coronary Syndromes	Kidney allograft rejection
Acute graft-versus-host disease	Leprosis
Acute kidney injury	Lupus nephritis
Acute-on-chronic liver failure	Malaria
Alcoholic hepatitis	Measles
Asthma	Multiple sclerosis
Atherosclerosis	Non-alcoholic steatohepatitis
Atrial fibrillation	Osteoarthritis
Celiac disease	Proliferative Diabetic Retinopathy
Chronic heart failure	Psoriasis
Chronic viral hepatitis	Rheumatoid arthritis
Cirrhosis	Sarcoidosis
COVID-19	Scleroderma
Crohn’s disease	Sepsis
Dengue	Sickle cell disease
EBV infection (Epstein–Barr virus infection)	Spondyloarthropathy
Gestational diabetes mellitus	Systemic lupus erythematosus
Hantaan virus	Systemic sclerosis
HBV (Hepatitis B virus), HCV (Hepatitis C virus)	Type 1 diabetes mellitus
Hemophagocytic lymphohistiocytosis	Type 2 diabetes mellitus
HIV (Immunodeficiency Virus)	Ulcerative colitis
Influenza	Visceral Leishmaniasis
